# Antibacterial and Anti-Efflux Activities of Cinnamon Essential Oil against Pan and Extensive Drug-Resistant *Pseudomonas aeruginosa* Isolated from Human and Animal Sources

**DOI:** 10.3390/antibiotics12101514

**Published:** 2023-10-05

**Authors:** Mohamed A. I. Abdelatti, Norhan K. Abd El-Aziz, El-sayed Y. M. El-Naenaeey, Ahmed M. Ammar, Nada K. Alharbi, Afaf Alharthi, Shadi A. Zakai, Adel Abdelkhalek

**Affiliations:** 1 Department of Microbiology, Faculty of Veterinary Medicine, Zagazig University, Zagazig 44511, Egypt; AYEIneanaey@vet.zu.edu.eg (E.-s.Y.M.E.-N.); aaahmmad@vet.zu.edu.eg (A.M.A.); 2 Department of Biology, College of Science, Princess Nourah bint Abdulrahman University, Riyadh 11671, Saudi Arabia; nkalharbi@pnu.edu.sa; 3 Department of Clinical Laboratory Sciences, College of Applied Medical Sciences, Taif University, Taif 21944, Saudi Arabia; a.awwadh@tu.edu.sa; 4 Department of Clinical Microbiology and Immunology, Faculty of Medicine, King Abdulaziz University, Jeddah 21589, Saudi Arabia; szakai@kau.edu.sa; 5 Food Safety, Hygiene and Technology Department, Faculty of Veterinary Medicine, Badr University in Cairo (BUC), Badr City 11829, Egypt; adel.abdelkhalek@buc.edu.eg

**Keywords:** *Pseudomonas aeruginosa*, efflux pumps, cinnamon oil, *MexB* and *MexA* genes, antimicrobial activity

## Abstract

*Pseudomonas aeruginosa* is notorious for its ability to develop a high level of resistance to antimicrobial agents. Resistance-nodulation-division (RND) efflux pumps could mediate drug resistance in *P. aeruginosa*. The present study aimed to evaluate the antibacterial and anti-efflux activities of cinnamon essential oil either alone or combined with ciprofloxacin against drug resistant *P. aeruginosa* originated from human and animal sources. The results revealed that 73.91% of the examined samples were positive for *P. aeruginosa*; among them, 77.78% were of human source and 72.73% were recovered from animal samples. According to the antimicrobial resistance profile, 48.73% of the isolates were multidrug-resistant (MDR), 9.2% were extensive drug-resistant (XDR), and 0.84% were pan drug-resistant (PDR). The antimicrobial potential of cinnamon oil against eleven XDR and one PDR *P. aeruginosa* isolates was assessed by the agar well diffusion assay and broth microdilution technique. The results showed strong antibacterial activity of cinnamon oil against all tested *P. aeruginosa* isolates with inhibition zones’ diameters ranging from 34 to 50 mm. Moreover, the minimum inhibitory concentration (MIC) and minimum bactericidal concentration (MBC) values of cinnamon oil against *P. aeruginosa* isolates ranged from 0.0562–0.225 µg/mL and 0.1125–0.225 µg/mL, respectively. The cinnamon oil was further used to evaluate its anti-efflux activity against drug-resistant *P. aeruginosa* by phenotypic and genotypic assays. The cartwheel test revealed diminished efflux pump activity post cinnamon oil exposure by two-fold indicating its reasonable impact. Moreover, the real-time quantitative polymerase chain reaction (RT-qPCR) results demonstrated a significant (*p* < 0.05) decrease in the expression levels of *MexA* and *MexB* genes of *P. aeruginosa* isolates treated with cinnamon oil when compared to the non-treated ones (fold changes values ranged from 0.4204–0.7474 for *MexA* and 0.2793–0.4118 for *MexB*). In conclusion, we suggested the therapeutic use of cinnamon oil as a promising antibacterial and anti-efflux agent against drug-resistant *P. aeruginosa*.

## 1. Introduction

*Pseudomonas aeruginosa* is a highly adaptive and robust organism. It can thrive in a wide range of environmental niches owing to its large and dynamic genome that provides extraordinary metabolic versatility and genetic plasticity [1]. *P. aeruginosa* is an opportunistic, Gram-negative bacillus that causes a variety of clinically important infections in compromised and critically ill individuals. It is commonly involved in patients with cystic fibrosis, severe burns, neutropenia, cancer, chronic obstructive pulmonary disorder (COPD), and severe infections necessitating ventilation, such as Coronavirus disease 2019 (COVID-19) [2]. *P. aeruginosa* causes a great variety of acute and chronic infections with high morbidity and mortality levels. These infections are particularly difficult to eradicate due to the expression of various virulence factors, the intrinsic antimicrobial resistance, and the owing ability to acquire resistance to numerous antimicrobial classes during therapy, which eventually leads to treatment failure [3]. The expression of multidrug efflux pumps within the resistance-nodulation-cell division (RND) family is largely responsible for *P. aeruginosa*’s inherent resistance [4,5]. These pumps are membrane proteins that are chromosomally encoded. They form tripartite complexes that include an outer-membrane channel protein, an inner membrane transporter protein, and a periplasmic adapter protein [6,7]. Together, these proteins create an efflux pump with a high level of efficiency that can remove a variety of structurally unrelated antimicrobial drugs from the cell [8]. However, the acquired resistance includes the development of resistance genes or mutations in the genes that code for penicillin-binding proteins, efflux pumps, porins, and chromosomal β-lactamase, all of which contribute to resistance to fluoroquinolones, aminoglycosides, β -lactams, and carbapenems [9]. A genomic study has discovered structural genes for at least twelve RND-type efflux systems, four of which have been demonstrated to play a role in multidrug resistance (MDR) (MexAB-OprM, MexCD-OprJ, MexEF-OprN, and MexXY-OprM) [10]. Of all, *P. aeruginosa* RND pumps, MexAB-OprM has the widest range of substrates and can pump out a variety of antimicrobials relating to different classes including β-lactams (carboxypenicillins, extended spectrum cephalosporins, aztreonam, and carbapenems such as meropenem and panipenem except imipenem and biapenem), tetracyclines, fluoroquinolones, macrolides, chloramphenicol, trimethoprim, sulfonamides, and novobiocin. It is produced constitutively in wild-type cells, and the overexpression of this specific pump can result in the MDR pattern [11]. Overexpression of the MexAB-OprM efflux pump is caused by the exposure to specific substrates or stressors (mostly through mutational alterations in the regulatory genes such as *mexR*, *nalC*, or *nalD*) [12]. Because of its efflux pump’s versatile substrate profile, the accumulation of many different substances such as antibiotics, disinfectants, detergents, and dyes will be reduced, resulting in MDR *P. aeruginosa* [13,14]. Ironically, very few effective antibiotics are available for treating *P. aeruginosa* infections, and in some cases, none at all [15]. All this necessitates the urgent need for fresh approaches to develop a new bactericidal to replenish the otherwise drying arsenal of anti-infective agents against drug-resistant *P. aeruginosa* strains [16].

As the active efflux of antibacterial drugs plays a substantial role in bacterial drug resistance, inhibiting efflux pumps looks to be a promising technique for restoring antibacterial efficacy. Therefore, the efflux pumps have been selected as possible targets for the development of therapeutic strategies using combinations of antibiotics and efflux pump inhibitors (EPIs) to restore the antibiotics efficiency [17]. Many EPIs of synthetic and natural origins have been discussed in the literature; however, none of them have entered clinical trials to date [18]. Natural products can be acquired from a variety of sources, including bacteria, fungi, algae, plants, and animals, but there is a growing interest in the bioactive substances produced by plants [19]. The essential oils account for a source of very promising natural compounds that can achieve efficient control of the antibiotic-resistant microorganisms [20,21]. Numerous studies have reported strong antibacterial activities of some essential oils, and their roles in efflux pumps inhibition [22]. The potential antibacterial effect of cinnamon oil has been documented frequently, but its activity against extensive drug-resistant (XDR) and pan drug-resistant (PDR) *P. aeruginosa* isolates is scare [23,24]. Therefore, we evaluated the antibacterial activity of cinnamon oil either alone or combined with ciprofloxacin against drug resistant *P. aeruginosa* (especially XDR and PDR). Thereafter, the anti-efflux potential of the subinhibitory concentration (SIC or SUB-MIC) of the essential oil against XDR and PDR *P. aeruginosa* isolates was investigated for the first time.

## 2. Results

### 2.1. Occurrence of P. aeruginosa in Animal and Human Samples

In all, 119 out of 161 (73.91%) examined samples were positive for *P. aeruginosa*. Out of 27 samples of human origin, 21 (77.78%) were positive, with the highest isolation rate from human burns (13 out of 16; 81.25%), followed by urine samples (8 out of 11; 72.73%). Regarding animal samples, *P. aeruginosa* was isolated from 80 out of 110 (72.73%) examined poultry samples with a high isolation rate from chicken liver (81.81%), followed by cloacal swabs (75%), lung and trachea (73.33% each), chicken heart (66.66%) and cecal parts (65.21%). While the existence of *P. aeruginosa* in mastitis milk was 75% (18 out of 24) (Table 1). Statistical analysis revealed a non-significant variation in the occurrence of *P. aeruginosa* in animal and human sources (*p* > 0.05).

Phenotypic identification of *P. aeruginosa* revealed Gram-negative bacilli arranged either singly or in groups, non-spore forming and non-capsulated. On pseudomonas agar media, *Pseudomonas* species appeared as circular, raised with an undulated margin surrounded by a blue to green zone due to pyocyanin formation. Biochemical series could identify *P. aeruginosa* simply. *P. aeruginosa* isolates were oxidase, catalase, and citrate tests positive. On TSI agar media, the expected results were alkaline slant (red) and alkaline butt (red). *P. aeruginosa* isolates were further confirmed by PCR-based detection of the genus (16S rRNA) and species-specific (*oprL*) genes, giving amplicons of 618 and 504 bp, respectively.

### 2.2. Antimicrobial Susceptibilities of P. aeruginosa Isolates

The antimicrobial susceptibilities of 119 *P. aeruginosa* isolates against 17 tested antimicrobial agents demonstrated that all isolates were resistant to fosfomycin. Moreover, high resistance rates were observed against polymixin B and colistin (74.78% each), followed by ceftazidime and cefepime (38.65% each). On the other hand, our results showed that ticarcillin-clavulanic acid (6.72%), piperacillin-tazobactam (5.88%) doripenem and meropenem (5.04% each) and imipenem (4.2%) had the lowest resistance rates against the tested isolates (Table 2). Statistical analysis showed non-significant differences (*p* > 0.05) in the antimicrobial susceptibilities of *P. aeruginosa* isolates to all tested antimicrobials except for carbapenems and polypeptides (*p* < 0.05).

According to the antimicrobial resistance profile, 48.73% (*n* = 58) of the isolates were MDR (MAR index = 0.23– 0.58) and 9.2% (*n* = 11) were XDR (MAR index = 0.58–0.88), of which eight isolates originated from poultry, only two were of human origin and one isolate was originated from cattle. Interestingly, one (0.84%) *P. aeruginosa* isolate originated from a chicken cloacal swab was PDR (MAR index = 1) (Table 2). Of note, nine XDR rather than the PDR *P. aeruginosa* isolates (10/12 = 83.33%) were CIP-resistant (MIC = 4–128 µg/mL).

### 2.3. Chemical Composition of Cinnamon Essential Oil

Gas Chromatography-Mass Spectrometry (GC-MS) analysis resulted in the identification of five chemical compounds for cinnamon essential oil, as indicated in Table 3. Cinnamaldehyde is the major chemical compound (78.1%), followed by benzyl alcohol (16.67%), linalyl iso-valerate (2.6%), eugenol (1.5%), and β-caryophyllene (1.13%).

### 2.4. Antimicrobial Activity of Cinnamon Oil and Ciprofloxacin against XDR and PDR P. aeruginosa Isolates

The antimicrobial potential of cinnamon oil against eleven XDR and one PDR *P. aeruginosa* isolates was assessed by the agar well diffusion assay and broth micro-dilution technique. The results revealed strong antimicrobial activity of cinnamon oil against all tested *P. aeruginosa* isolates with inhibition zones’ diameters ranging from 34 to 50 mm. Moreover, the MIC and MBC values of cinnamon oil against tested isolates ranged from 0.0562– 0.225 µg/mL and 0.1125–0.225 µg/mL, respectively. The MIC90 and MIC50 of cinnamon oil were the same value (0.25 µg/mL). On the other hand, ciprofloxacin showed lower antimicrobial activity against tested *P. aeruginosa* isolates (2–256 µg/mL). MIC90 and MIC50 of ciprofloxacin against tested isolates were 128 and 32 µg/mL, respectively.

**Figure 1:**
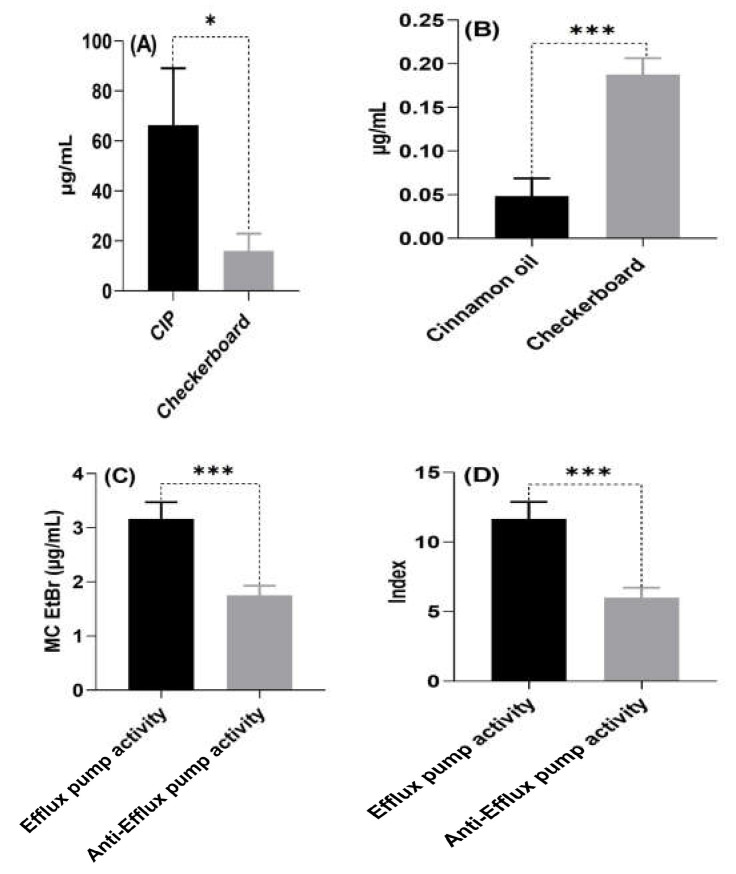
Comparison of checkerboard result vs. both of ciprofloxacin (**A**) and cinnamon oil (**B**), efflux pump activity vs. anti-Efflux pump activity for both of MC EtBr (**C**) and index (**D**). CIP, ciprofloxacin, MC EtBr, minimum EtBr concentration. * Statistically significant at *p*-value < 0.05, *** highly significant at *p*-value < 0.001.

The results of ΣFIC of the checkerboard assay showed synergistic antimicrobial interactions of cinnamon oil and ciprofloxacin against 10 out of 12 tested *P. aeruginosa* isolates (83.33%). The MIC results of both cinnamon oil and ciprofloxacin together decreased when compared with the MIC results of each alone. The MIC values of ciprofloxacin reduced by one–sixfold in the presence of cinnamon oil in six out of ten (60%) ciprofloxacin-resistant isolates. However, the synergistic ciprofloxacin/cinnamon oil combination resulting in switching of only two out of ten (20%) ciprofloxacin resistant isolates to be sensitive (MIC values = 128 → 2 and 32 → 1 µg/mL). Significant differences were observed between the MIC values of the checkerboard assay for ciprofloxacin and cinnamon oil (*p* = 0.0005, and 0.0001, respectively) (Figure 1A,B). As the best antibacterial activity was shown by cinnamon oil, it was further used to evaluate its anti-efflux activity against drug-resistant *P. aeruginosa* isolates by phenotypic and genotypic assays.

### 2.5. Determination of the Efflux Pump’s Activity Phenotypically

In order to measure the efflux activity of XDR (*n* = 11) and PDR (*n* = 1) *P. aeruginosa* isolates, the bacteria’s capacity to expel the EtBr out of the cell was detected using the cartwheel method. It was found that *P. aeruginosa* isolates fluoresced when they developed in a confluent mass along a radial line of TSA plates with increasing EtBr concentrations. Table 4 illustrates the minimal EtBr concentration and efflux activity index for each isolate of *P. aeruginosa*. At an EtBr concentration of 4 g/mL, seven isolates (7/12; 58.33 %) started to fluoresce, while the rest of isolates (5/12; 41.67%) fluoresced at EtBr concentrations below 4 g/mL.

The anti-efflux activities of cinnamon oil against drug-resistant *P. aeruginosa* isolates are shown in Table 4. Statistical analysis demonstrated that the MC EtBr and index differ significantly pre- and post-treatment with cinnamon essential oil (*p* < 0.0001 and 0.0005, respectively) (Figure 1C,D).

### 2.6. Quantification of the Expression Levels of Efflux Pump Genes Using RT-qPCR

**Figure 2:**
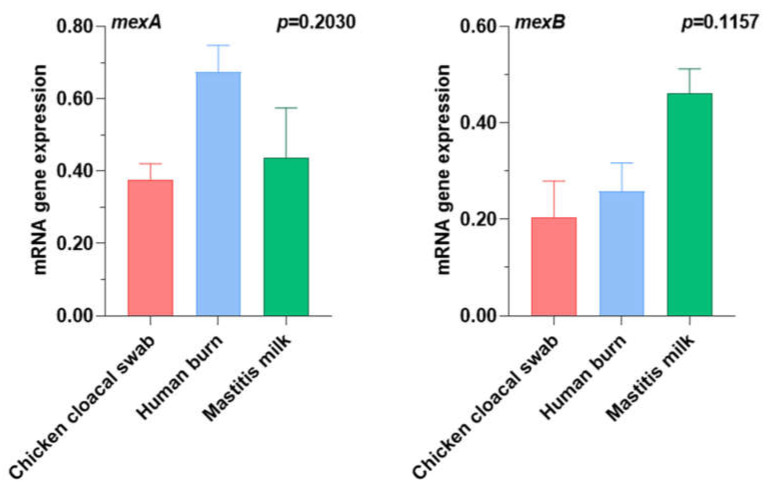
Relative expression of the efflux pump genes post treatment with cinnamon essential oil in drug resistant *P. aeruginosa* isolates. As presented in Table 4, chicken cloacal swab code no.9, human burn code no. 11 and mastitis milk code no. 12.

To further confirm the inhibitory effects of the SIC of cinnamon oil on the efflux pump activity of drug-resistant *P. aeruginosa* isolates reported here (*n* = 3; code no. 9, 11, and 12 in Table 4), the transcript levels of efflux-associated genes; *MexA* and *MexB* genes, and their regulator (*oprL*) were determined by reverse transcriptase quantitative polymerase chain reaction (RT-qPCR) in triplicate (Supplementary Materials Figure S1). Data analysis indicated that *P. aeruginosa* isolates showed low transcript levels of the efflux-associated genes, *MexA* (fold change range = 0.4204–0.7474) and *MexB* (fold change range = 0.2793–0.4118), in all treatments when compared to the untreated isolates (Figure 2). The current findings revealed notable non-significant differences in the expression levels of the two efflux pump genes under investigation (*p* = 0.2030 and 0.1157 for *MexA* and *MexB*, respectively) across various sources including chicken cloacal swab, human burn, and mastitis milk.

## 3. Discussion

*Pseudomonas aeruginosa*, a leading nosocomial pathogen, is responsible for healthcare-associated infections. It has not only emerged as a MDR pathogen but evolved as an XDR and a PDR as well [25]. There are currently no new antimicrobials that can be used to treat these bacteria in place of the ones that already exist, and there is no widely accessible vaccination against such infections. The strategy to lessen the negative consequences is to control the spread of infections and dissemination of antimicrobial resistance [26,27], which could be achieved through understanding the dynamics, causes, and difficulty of the organism prevalence. Drug resistance evolution has drawn attention to conventional therapies, including herbal remedies. Both humans and animals around the world have received therapy for a variety of infectious ailments with natural alternatives [28]. The objective of the present study was to assess the antibacterial potential of cinnamon essential oil in combination with ciprofloxacin against drug resistant *P. aeruginosa*. Furthermore, the anti-efflux activity of cinnamon oil against XDR and PDR *P. aeruginosa* isolated from human and animal sources by phenotypic and genotypic assays.

Herein, the overall prevalence rate of *P. aeruginosa* was 73.91%, representing 77.78% from human, 72.73% from poultry, and 75% from mastitic milk. In the literature, nosocomial strains of *P. aeruginosa* appear to be more common everywhere, particularly as a cause of ventilator-associated pneumonia; they are also becoming more common in high-risk populations, such as patients with severe burn injuries [29]. *Pseudomonas* predominated the respiratory tract (42.8%), followed by wounds (skin/soft tissue, 26%), urine (13.5%) and blood cultures (6.5%) [30], which is lower than our results where the highest existence of the microorganism was in human burns (81.25%), followed by urine samples (72.73%). Furthermore, Mahmoud et al. stated that *P. aeruginosa* accounted for 32.3 % of infections in burn unit at Menofia University Hospitals [31].

For the purpose of determining the prevalence of *P. aeruginosa* infection among various flocks of chickens, various surveillance studies have been carried out. For instance, Shukla and Mishra [32] isolated *P. aeruginosa* from healthy chicks at a rate of 12% and from diseased ones at a rate of 30%. Furthermore, *P. aeruginosa* was recovered from broiler chicken flocks with low percentages; 21.6% [33] and 17.6% [34] from different Egyptian governorates. Moreover, *P. aeruginosa* has been detected in 42 of 480 (8.75%) broiler chicken samples [35]. Conversely, 32 of 46 broiler chicken farms (69.57%) were positive for *P. aeruginosa* [36], which is in line with our results.

*P. aeruginosa* is an environmentally abundant bacterium that causes severe diseases among immune compromised hosts. It is one of the common causes of mastitis [37]. Ibrahim and co-workers could isolate the pathogen from mastitic animals by a lower percentage (34%) [38]. The difference in isolation rates may be attributed to geographical areas, climatic circumstances, sample types, stress factors, and growth conditions.

*P. aeruginosa* strains are frequently and intrinsically resistant to a broad range of antibiotics. In this study, analysis of the antimicrobial resistance of *P. aeruginosa* isolates demonstrated absolute resistance for fosfomycin (100%), followed by polymixin B and colistin (74.78%). Whereas ticarcillin-clavulanic acid (6.72%), piperacillin-tazobactam (5.88%) doripenem and meropenem (5.04% each) and imipenem (4.2%) had the lowest resistance rates against the tested isolates. In contrast, Dorri and coworkers reported 100% susceptibility of *P. aeruginosa* isolates to colistin and polymixin B [39]. This variation in resistance may be related to the habitual utilization of certain antibiotics for the treatment of numerous diseases in various geographical regions. The overuse of antibiotics has led to the emergence of MDR strains; therefore, the first step in preventing the spread of antibiotic resistance is to continue reporting the resistance rates. Here, 48.73% of *P. aeruginosa* isolates were MDR, with alarming increase in XDR (9.2%) and PDR (0.84%) categories. Previous studies showed varies trends from other countries. Our MDR percentage was high in comparable to previous researches conducted by Gad et al. [40] in Egypt (36%) and Sabir et al. [41] in Pakistan (22.08%). On the contrary, Tartor and coauthors [42] in Egypt (100%), Inan et al. [43] in Turkey (60%) and Gill team [25] in India (50%) have reported a high prevalence of MDR *P. aeruginosa* isolates. However, 2.3% XDR and no PDR phenotypes were previously recorded [25].

*P. aeruginosa* is an obstinate microorganism in terms of resistance to various antimicrobials and possesses three main mechanisms of limited adsorption resistance and efflux, drug inactivation, and change in targets [44]. Efflux pumps play a key role in *P. aeruginosa* resistance. The ejection of hazardous chemicals and decreased antibiotic sensitivity are both caused by *P. aeruginosa*’s multidrug efflux pumps [45]. In addition, overexpression of these pumps in *P. aeruginosa* is directly linked to resistance to the majority of anti-pseudomonal remedies and may impair the effectiveness of novel types of anti-pathogen medications. The operon MexAB-OprM is regarded as the primary cause of antibiotic resistance and was the first multidrug efflux pump discovered in *P. aeruginosa* [46]. Quinolones, beta-lactams, and a wide variety of other anti-microorganisms are excreted by it.

In this study, cinnamon oil was examined against 12 drug-resistant *P. aeruginosa* isolates. It showed a strong antimicrobial activity against all tested isolates exhibiting inhibition zones ranging from 34–50 mm in diameter on agar well diffusion assay and low MIC and MBC values (0.0562–0.225 µg/mL and 0.1125–0.225 µg/mL, respectively) by the broth microdilution technique. These results were consistent with the study conducted by Utchariyakiat et al. in which cinnamon oil demonstrated the most inhibitory effectiveness against MDR *P. aeruginosa* clinical isolates with MIC range of 0.1125–0.225 µg/mL [47].

In this situation, combining essential oils with antibiotics may have a synergistic antibacterial effect, resulting in the creation of a novel treatment strategy. Ciprofloxacin demonstrated adequate antibacterial efficacy in this instance against the tested *P. aeruginosa* isolates (MIC range = 2–256 µg/mL). Limited reports have conducted on the combination of cinnamon with various antimicrobials or nanoparticles and the results demonstrated synergistic or additive effects against various MDR microorganisms [47,48,49,50]. In accordance with a previous study [47], the combination of cinnamon oil with ciprofloxacin showed partial synergism against clinical *P. aeruginosa* isolates. In 2012, Guerra et al. published an investigation on the antibacterial activity of *Cinnamon zeylanicum* essential oil in combination with gentamicin and amikacin against *Actinobacter* species showing additive and synergistic activities, respectively [51]. Furthermore, Yap et al. reached similar results, where the combination of cinnamon bark essential oil and piperacillin induced a considerable reduction in the registered MIC values of piperacillin presenting a synergistic effect against a clinical strain of beta-lactamase-producing *E. coli* [49]. Mahadlek et al. used the checkerboard assay to determine the activity of cinnamon oil associated with doxycycline hyclate, ciprofloxacin HCl and metronidazole. They observed an additive activity of cinnamon oil combinations with doxycycline hyclate, ciprofloxacin HCl, or metronidazole against *S. aureus* ATCC 6538P [48]. Thus, it could be possible that cinnamon oil may form complexes with ciprofloxacin that may increase its antibacterial activity. Moreover, cinnamon oil could also inhibit the efflux transporters, which leads to a rise in the efficacy of antibiotics against tested isolates.

In the present study, the expression of *MexA* and *MexB* genes before and after contact with efflux pump inhibitors (cinnamon oil) were investigated by the RT-qPCR technique. The relative expression of *MexA* and *MexB* genes in cinnamon oil-treated isolates were significantly more reduced than the non-treated ones (fold changes values ranged from 0.4204–0.7474 for *MexA* and 0.2793–0.4118 for *MexB*). According to the findings, the inhibitory effect of cinnamon oil on the *MexB* gene was higher than that of the *MexA* one. On the other hand, a previous study conducted in France demonstrated that using cinnamon bark oil or cinnamaldehyde as an additional therapy to treat *P. aeruginosa* infections could have antagonistic effects when taken with antibiotics. According to their explanation, Mex pump activation strongly increased the expression of operons that code for efflux systems MexXY/OprM, MexAB-OprM, MexCD-OprJ, and MexEF-OprN [52]. However, similar data were not available for comparison with other authors about the anti-efflux activity of cinnamon oil in PDR *P. aeruginosa* bacterial pathogen.

## 4. Materials and Methods

### 4.1. Sampling

One hundred and sixty-one samples were collected during the period between November 2020 to April 2022 from both animal (*n* = 134) and human (*n* = 27) origins. Animal samples included chicken organs (*n* = 110 comprising liver (22), heart (15), cloacal swabs (20), cecal contents (23), lung and trachea (30)), and mastitis milk (*n* = 24), which were collected from sporadic cases of mastitic dairy cows at Zagazig City, Sharkia Governorate, Egypt. Human samples included burns (*n* = 16) and urine (*n* = 11), those were collected from patients attending various hospitals and laboratories at Hehia City, Sharkia Governorate, Egypt. The samples were put aseptically into sterile containers, kept in an icebox, and transferred as soon as possible to the Bacteriology Laboratory, Department of Microbiology, Faculty of Veterinary Medicine, Zagazig University, for further examination. The study was approved by Zagazig University Institutional Animal Care and Use Committee (ZU-IACUC) (approval number ZU-IACUC/2/F/404/2022). Written informed consent was obtained from the owners for the participation of their animals in this study. The patients/participants provided their written informed consent to participate in this study.

### 4.2. Isolation and Identification of P. aeruginosa

For the isolation of *P. aeruginosa*, swabs samples were enriched in brain heart infusion broth (BHI; Oxoid, Hampshire, UK) then they were promptly cultivated onto pseudomonas agar base selective medium supplemented with pseudomonas selective supplements (Oxoid, Hampshire, UK). The colonial pigmentation and conventional biochemical assays involving oxidase, catalase, and citrate tests and the biochemical reactions on triple sugar iron (TSI, Oxoid, Hampshire, UK) agar presumptively identified the detected colonies as *P. aeruginosa* using standard microbiological techniques [53]. Genus- and species-specific oligonucleotide primers (Metabion, Planegg, Germany) were used for the identification of 16S rRNA and *oprL* genes, respectively [54]. All the isolates were stored frozen at −20 °C in individual aliquots in BHI broth with 25% glycerol until further analysis.

### 4.3. Antimicrobial Susceptibility Testing of P. aeruginosa Isolates

The antimicrobial susceptibilities of *P. aeruginosa* were evaluated against 17 commercially available antimicrobial agents representing eight different classes (Oxoid, Hampshire, UK) using the disk diffusion method [55]. The tested antimicrobials were gentamicin (GEN, 10 µg), tobramycin (TOB, 10 µg), amikacin (AK, 30 µg), netilmicin (NET, 30 µg), imipenem (IPM, 10 µg), meropenem (MRP, 10 µg), doripenem (DOR, 10 µg), cefepime (FEP, 30 µg), ceftazidime (CAZ, 30 µg), ciprofloxacin (CIP, 5 µg), levofloxacin (LEV, 5 µg), ticarcillin-clavulanic acid (TIC, 100/10 µg), piperacillin-tazobactam (PTZ,100/10 µg), fosfomycin (FF, 200 µg), aztreonam (ATM, 30 µg), polymyxin B (PB, 300 U) and colistin (CT, 10 µg). The inhibition zone diameters were measured and interpreted according to Clinical and Laboratory Standards Institute (CLSI) and European Committee on Antimicrobial Susceptibility Testing (EUCAST) guidelines [56,57]. The MDR was identified as acquired resistance of a microorganism to at least one antibiotic in three or more antimicrobial categories. Extensively drug-resistance (XDR) was identified as resistance of a single bacterium to all antibiotics except two or fewer antimicrobial categories, whereas pan drug-resistance (PDR) was identified as resistance of a microorganism to all antibiotics in all antimicrobial categories [58]. Each isolate’s multiple antibiotic resistance (MAR) index was determined as follows: number of antimicrobials that the isolate was resistant to the number of antimicrobials that the isolate had been tested; while the MAR index for each antimicrobial is calculated as follows: total number of resistance obtained/(total numbers of tested antimicrobials × total number of isolates) [59].

### 4.4. Cinnamon Oil

A stock solution of ≤ 100% commercially available cinnamon oil (Sigma, Berlin, Germany) was prepared in tryptic soy broth (TSB; Oxoid, Hampshire, UK) containing 1% (*v*/*v*) dimethylsulfoxide (DMSO; Sigma Aldrich, Seelze, Germany). Preliminary testing revealed that 1% DMSO in the final concentration did not demonstrate antimicrobial activity.

### 4.5. Characterization of Cinnamon Essential Oil by Gas Chromatography-Mass Spectrometry

Gas Chromatography-Mass Spectrometry (GC-MS system; Agilent Technologies, Santa Clara, CA, USA) was equipped with gas chromatograph (GC; 7890B) and mass spectrometer detector (5977A) at Central Laboratories Network, National Research Centre, Cairo, Egypt. Samples were diluted with hexane (1:19, *v*/*v*). The GC was equipped with HP-5MS column ((5%-phenyl)-methylpolysiloxane, 30 m × 0.25 mm internal diameter and 0.25 μm film thickness). Analyses were carried out using helium as the carrier gas at a flow rate of 1.0 mL/min at a split ratio of 1:10, injection volume of 1 µL and the following temperature program: 40 °C for 1 min; rising at 4 °C/min to 150 °C and held for 6 min; rising at 4 °C/min to 210 °C and held for 5 min. The injector and detector were held at 280 °C and 220 °C, respectively. Mass spectra were obtained by electron ionization (EI) at 70 eV; using a spectral range of *m*/*z* 50–550 and solvent delay of 3 min. Identification of different constituents was determined by comparing the spectrum fragmentation pattern with those stored in Wiley and National Institute of Standards and Technology (NIST) mass spectral library data [60].

### 4.6. Antimicrobial Activities of Cinnamon Oil and Ciprofloxacin against P. aeruginosa Isolates

The antimicrobial activities of cinnamon oil (100%) were determined against drug-resistant *P. aeruginosa* isolates. The agar well diffusion method was performed following Valgas et al. [61] and the susceptible isolates exhibited inhibition zones’ diameters ≥ 8 mm as reported previously [62]. The minimum inhibitory concentrations (MICs) and minimum bactericidal concentrations (MBCs) of each antimicrobial agent were detected using the broth microdilution technique [63]. Moreover, the orderly array method [64] was adopted to calculate the MIC50 and MIC90 of the antimicrobials against tested isolates.

The interactions between cinnamon oil and ciprofloxacin were evaluated against *P. aeruginosa* isolates using the checkerboard method in 96-well microtiter plates [24]. In brief, eight two-fold serial dilutions of cinnamon oil and ciprofloxacin were made in Mueller Hinton broth (MHB; Oxoid, Hampshire, UK) in the grid of eight rows by eight columns. Ciprofloxacin was placed in the wells of eight rows in descending concentrations starting at two times the MICs. Cinnamon oil was similarly distributed among the eight columns. The last four columns of the microtiter plate served as controls for *P. aeruginosa* growth and plate sterility. An aliquot of 100 µL of *P. aeruginosa* (5 × 10^5^ CFU/mL) was added for each well. The plates were incubated at 37 °C for 24 h. The analysis of the combination was obtained by calculating the fractional inhibitory concentration index (FICI) [65] using the following formula:

The FICI = FICA + FICB;
FICA = MIC of A in combination/MIC of A alone;
FICB = MIC of B in combination/MIC of B alone.

Where A is cinnamon essential oil and B is ciprofloxacin. Interpretation of the FICI was as follows: synergistic effect ≤ 0.5; partial synergy > 0.5 to < 1; additive 1; indifference > 1 to < 4 and antagonism ≥ 4.

### 4.7. Phenotypic Detection of the Efflux Pump Activity by Ethidium Bromide Cartwheel (EtBr-CW) Method

By using the EtBr-CW method, the efflux pumps’ capacity to expel ethidium bromide was evaluated [66]. Concisely, freshly prepared trypticase soy agar (TSA; Oxoid, Hampshire, UK) plates containing ethidium bromide (EtBr; Sigma-Aldrich, Seelze, Germany) concentrations ranging from 0 to 4 mg/L (these concentrations were selected basing on the bacterial MICs of EtBr) were kept away from light on the day of the experiment. The tested bacterial isolates were grown in overnight cultures that were calibrated to a 0.5 McFarland turbidity standard (1.5 × 10^8^ CFU/mL). Ten to twelve performed sectors were arranged onto the 9 cm diameter TSA plates in a cartwheel pattern. On the EtBr-TSA plates, the adjusted bacterial cultures were swabbed from the plate’s center to its edge. The plates were inspected under an ultra-violet (UV) transilluminator (Cole-parmer, Vemon Hills, Chicago, IL, USA) after being incubated at 37 °C for 16 h. The smallest amount of EtBr required to obtain the bacterial mass to fluoresce was recorded. The isolates were classified as EtBr-CW-negative, EtBr-CW intermediate, or EtBr-CW-positive depending on whether they emitted fluorescence at 0.5–1 mg/L, 2 mg/L, or only 3–4 mg/L EtBr, respectively.

The capacity of each *P. aeruginosa* isolate to expel EtBr substrate was graded relative to the control isolate (a pan susceptible *P. aeruginosa* isolated during this study) according to the following equation: Efflux activity index = MCEtBr (XDR) − MCEtBr (Reference)/MCEtBr (Reference).

Where MCEtBr (XDR) represents the minimum EtBr concentration that produces fluorescence of the test isolate. Meanwhile, MCEtBr (Reference) indicates the minimum EtBr concentration that produces fluorescence of the control isolate.

### 4.8. Transcriptional Analysis of the Efflux Pump Genes Using Real-Time Quantitative PCR (RT-qPCR)

Total RNA was extracted from non-treated and treated *P. aeruginosa* isolates with the SICs (concentrations lower than the MIC values) of cinnamon oil in the logarithmic growth phase using QIAamp RNeasy Mini kit (Qiagen, Hilden, Germany) following the manufacturer’s instructions. The transcription analysis of *mexA* and *mexB* efflux pump genes was determined in triplicate by one-step RT-qPCR using QuantiTect SYBR Green RT-PCR Kit (Qiagen, Hilden, Germany) in the MX3005P real-time PCR thermal cycler (Stratagene, La Jolla, CA, USA) according to the manufacturer’s instructions. The oligonucleotide primer pairs and cycling conditions are listed in Table 5. RNA extraction from *P. aeruginosa* ATCC 27853 was used as a positive control, while nuclease-free water was used as a negative control. The specificity of the amplified products was verified by generating melting curves. The relative quantitation of mRNA expression of each sample was normalized to the constitutive expression of the *oprL* housekeeping gene. The fold changes in the transcript levels of targeted genes in treated *P. aeruginosa* relative to their levels in the untreated ones were calculated according to the comparative 2^−ΔΔCT^ method [67].

### 4.9. Statistical Analysis

Data were edited in Microsoft Excel (Microsoft Corporation, Redmond, WA, USA, accessed on 25 June 2023). The Levene and Shapiro–Wilk tests were used in order to check the normality and homogeneity of variance [71]. The differences between frequencies data were examined by fisher exact test according to the statistical analysis system [72]. The differences between means were assessed by Wilcoxon Signed-Ranks Test. Figures were fitted by the GraphPad Prism software 9.0 (GraphPad, La Jolla, CA, USA, accessed on 25 June 2023). Statistical significance was accepted as *p* < 0.05.

## 5. Conclusions

We provide a comprehensive overview of the antimicrobial and anti-efflux potential of cinnamon oil against PDR and XDR *P. aeruginosa* isolates for the first time. These findings highlight the promise of essential oils as a viable alternative for future dosing approaches to treat *P. aeruginosa* infections.

## Figures and Tables

**Table 1:** Occurrence of *P. aeruginosa* in animal and human samples.

Sample (No.)	No. of *P. aeruginosa* Isolates (%)	*p*-Value
Poultry samples (110)	80 (72.73)	
Cloacal swabs (20)	15 (75)	
Lung and trachea (30)	22 (73.33)	
Cecal contents (23)	15 (65.22)	0.7531
Chicken heart (15)	10 (66.67)	
Chicken liver (22)	18 (81.82)	
Mastitis milk (24)	18 (75)	
Human samples (27)	21 (77.78)	0.4281
Burn swabs (16)	13 (81.25)	
Urine (11)	8 (72.73)	
Total (161)	119 (73.91)	0.8591

**Table 2:** Resistance frequency of *P. aeruginosa* recovered from different sources.

Antimicrobial Class	Antimicrobial Agent	No. of Resistant *P. aeruginosa* Isolated from Different Sources (%)			No. (%)	*p*-Value
		Poultry (80)	Human (21)	Cattle (18)		
Aminoglycosides	Gentamicin	25 (31.25%)	7 (33.33%)	3 (16.67%)	35 (29.41%)	0.4281
	Tobramycin	25 (31.25%)	7 (33.33%)	3 (16.67%)	35 (29.41%)	0.4281
	Amikacin	25 (31.25%)	7 (33.33%)	3 (16.67%)	35 (29.41%)	0.4281
	Netilmicin	25 (31.25%)	7 (33.33%)	3 (16.67%)	35 (29.41%)	0.4281
Carbapenems	Imipenem	2 (2.5%)	3 (14.28%)	0 (0%)	5 (4.2%)	**0.0356**
	Meropenem	3 (3.75%)	3 (14.29%)	0 (0%)	6 (5.04%)	**0.0428**
	Doripenem	3 (3.75%)	3 (14.29%)	0 (0%)	6 (5.04%)	**0.0428**
Cephalosporin	Ceftazidime	27 (33.75%)	12 (57.14%)	7 (38.89%)	46 (38.66%)	0.1467
	Cefepime	27 (33.75%)	12 (57.14%)	7 (38.89%)	46 (38.66%)	0.1467
Fluoroquinolones	Ciprofloxacin	16 (20%)	2 (9.52%)	1 (5.56%)	19 (15.97%)	0.2150
	Levofloxacin	16 (20%)	2 (9.52%)	1 (5.56%)	19 (15.97%)	0.2150
Penicillin	Ticarcillin-clavulanic acid	6 (7.5%)	1 (4.76%)	1 (5.56%)	8 (6.72%)	0.8847
	Piperacillin-tazobactam	6 (7.5%)	1 (4.76%)	1 (5.56%)	8 (6.72%)	0.8847
Monobactam	Aztreonam	23 (28.75%)	15 (71.43%)	2 (11.11%)	40 (33.61%)	0.0001
Phosphonic acids	Fosfomycin	80 (100%)	21 (100%)	18 (100%)	119 (100%)	1.00
Polypeptide	Colistin	66 (82.5%)	9 (42.86%)	14 (77.78%)	89 (74.79%)	**0.0018**
	Polymyxin B	66 (82.5%)	9 (42.86%)	14 (77.78%)	89 (74.79%)	**0.0018**
MDR	-	38 (47.5%)	12 (57.14%)	8 (44.44%)	58 (48.74%)	0.6785
XDR	-	8 (10%)	2 (9.52%)	1 (5.56%)	11 (9.24%)	0.8401
PDR	-	1 (1.25%)	0 (0%)	0 (0%)	1 (0.84%)	0.7821

MDR, multidrug-resistance; XDR, extensive drug-resistance; PDR, pan drug-resistance. Bold values indicate significant differences at *p* < 0.05.

**Table 3:** Chemical constituents of cinnamon essential oil.

Compound	Retention Time (min)	Area under Peak	%
Benzyl alcohol	8.904	34162727.81	16.67
Linalyl iso-valerate	14.991	5320755.75	2.6
Cinnamaldehyde	15.557	160046038.4	78.1
Eugenol	17.781	3073334.82	1.5
β-Caryophyllene	19.421	2312309.18	1.13

**Table 4:** Phenotypic characterization of XDR *Pseudomonas* isolates.

Isolate No.	Source	Antimicrobial Resistant Pattern	MAR Index	MIC (µg/mL)		Checkerboard Result MIC (µg/mL)	Efflux Pump Activity		Anti-Efflux Pump Activity	
				CIP	Cinnamon Oil		MC EtBr (µg/mL)	Index	MC EtBr (µg/mL)	Index
1	Chicken cloacal swab	GEN, AK, NET, TOB, CAZ, FEP, PTZ, TIC, ATM, FF, PB, CT	0.70	2	0.25	0.5/0.0312	4	15	2	7
2	Chicken heart	GEN, AK, NET, TOB, CAZ, FEP, CIP, PTZ, TIC, ATM, FF, PB, CT	0.76	4	0.25	4/0.25	2	7	1	3
3	Chicken heart	GEN, AK, NET, TOB, CAZ, FEP, CIP, PTZ, TIC, ATM, FF, PB, CT	0.76	4	0.125	4/0.125	4	15	2	7
4	Chicken cecal content	GEN, AK, NET, TOB, CAZ, FEP, CIP, PTZ, TIC, ATM, FF, PB, CT	0.76	16	0.125	8/0.0156	2	7	1	3
5	Chicken cloacal swab	GEN, AK, NET, TOB, CAZ, FEP, CIP, LEV, ATM, FF, PB, CT	0.70	128	0.125	2/0.0078	4	15	2	7
6	Chicken liver	GEN, AK, NET, TOB, CAZ, FEP, CIP, LEV, ATM, FF, PB, CT	0.70	128	0.25	4/0.0312	4	15	2.5	9
7	Chicken liver	GEN, AK, NET, TOB, CAZ, FEP, CIP, LEV, ATM, FF, PB, CT	0.70	256	0.25	8/0.0312	4	15	2	7
8	Chicken cecal content	GEN, AK, NET, TOB, CAZ, FEP, CIP, IPM, MRP, DOR, PTZ, TIC, ATM, FF, PB, CT	0.94	64	0.25	64/0.0156	2.5	9	1.5	5
9	Chicken cloacal swab	GEN, AK, NET, TOB, IPM, MRP, DOR, CAZ, FEP, CIP, LEV, PTZ, TIC, ATM, FF, PB, CT	1	32	0.125	32/0.0156	4	15	2.5	9
10	Human burn	CAZ, FEP, CIP, LEV, PTZ, TIC, ATM, FF, PB, CT	0.58	32	0.125	1/0.0078	1.5	5	1	3
11	Human burn	GEN, AK, NET, TOB, IPM, MRP, DOR, CAZ, FEP, CIP, LEV, ATM, FF, PB, CT	0.88	128	0.25	64/0.0312	4	15	2.5	9
12	Mastitis milk	GEN, AK, NET, TOB, CAZ, FEP, PTZ, TIC, ATM, FF, PB, CT	0.70	2	0.125	1/0.0156	2	7	1	3
Mean ± SE				66.333 ± 22.751 *	0.187 ± 0.018 ^Π^	16.041 ± 6.914/ 0.048 ± 0.020	3.166 ± 0.303 ^¶^	11.667 ±1.214 ^╥^	1.75 ± 0.179	6.00 ± 0.717

MIC, minimum inhibitory concentration; GEN, gentamicin; AK, amikacin; NET, netilmicin; TOB, tobramycin; IPM, imipenem; MRP, meropenem; DOR, doripenem CAZ, ceftazidime; FEP, cefepime CIP, ciprofloxacin LEV, levofloxacin; PTZ, ticarcillin-clavulanic acid; TIC, piperacillin-tazobactam; ATM, aztreonam FF, fosfomycin; PB, polymyxin B; CT, colistin; MAR, multiple antibiotic resistance; MIC, minimum inhibitory concentration; MC EtBr, minimum EtBr concentration; SE, standard error *, ^Π^, ^¶^, ^╥^ differ significantly with checkerboard, MC EtBr, and Index, respectively. All isolates were XDR except the isolate No. 9 was PDR.

**Table 5:** Oligonucleotide primers used in the study.

Target Gene	Primers Sequences 5 → 3′	Specificity	Annealing Temperature (° C)	Product Size (bp)	References
*16S rRNA*	F: GACGGGTGAGTAATGCCTA R: CACTGGTGTTCCTTCCTATA	*Pseudomonas* species	54	618	[54]
*oprL*	F: ATGGAAATGCTGAAATTCGGC R: CTTCTTCAGCTCGACGCGACG	*P. aeruginosa* and an internal control	57	504	[68,69]
*mexA*	F:ACCTACGAGGCCGACTACCAGA R: GTTGGTCACCAGGGCGCCTTC	Efflux pump gene	61	179	[70]
*mexB*	F: GTGTTCGGCTCGCAGTACTC R: AACCGTCGGGATTGACCTTG	Efflux pump gene		244	

F, forward; R, reverse; bp, base pair.

## Data Availability

The data presented in this study are available on request from the corresponding author.

## Supplementary Materials

The following supporting information can be downloaded at: https://www.mdpi.com/article/10.3390/antibiotics12101514/s1, Figure S1: Data sheet of RT-qPCR showing amplification curves of *MexA* (A) *MexB* (B) and *OprL* (C) genes of *P. aeruginosa*.
