# Supplementary material for: Antibacterial and Anti-Efflux Activities of Cinnamon Essential Oil against Pan and Extensive Drug-Resistant Pseudomonas aeruginosa Isolated from Human and Animal Sources
Source: Antibiotics (Basel). 2023 Oct 5;12(10):1514. doi: 10.3390/antibiotics12101514 (PMC10604284; doi:10.3390/antibiotics12101514)
Supplement: Supplementary file 1 [file antibiotics-12-01514-s001.zip › antibiotics-2561636-supplementary.pdf]

Supplementary Materials

(A)

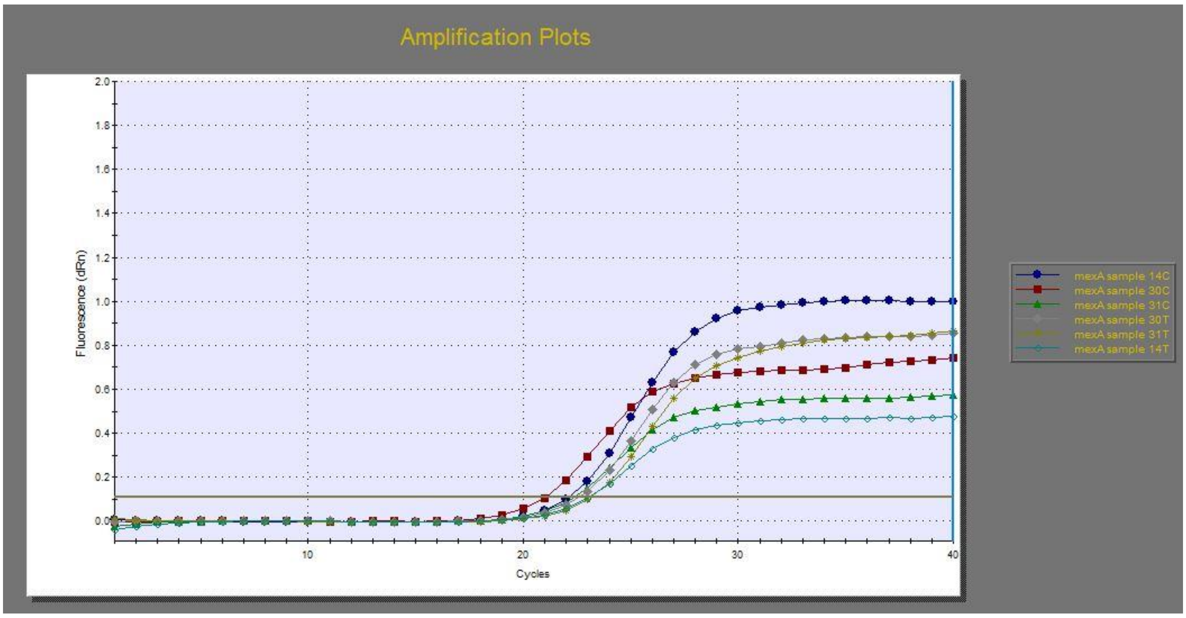

(B)

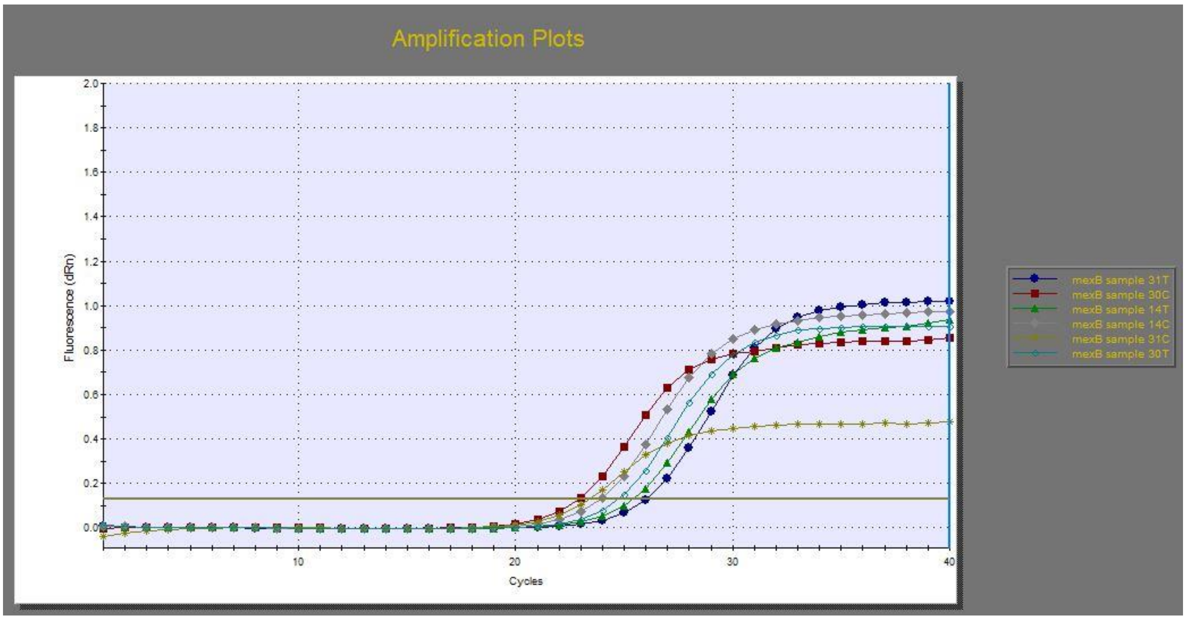

(C)

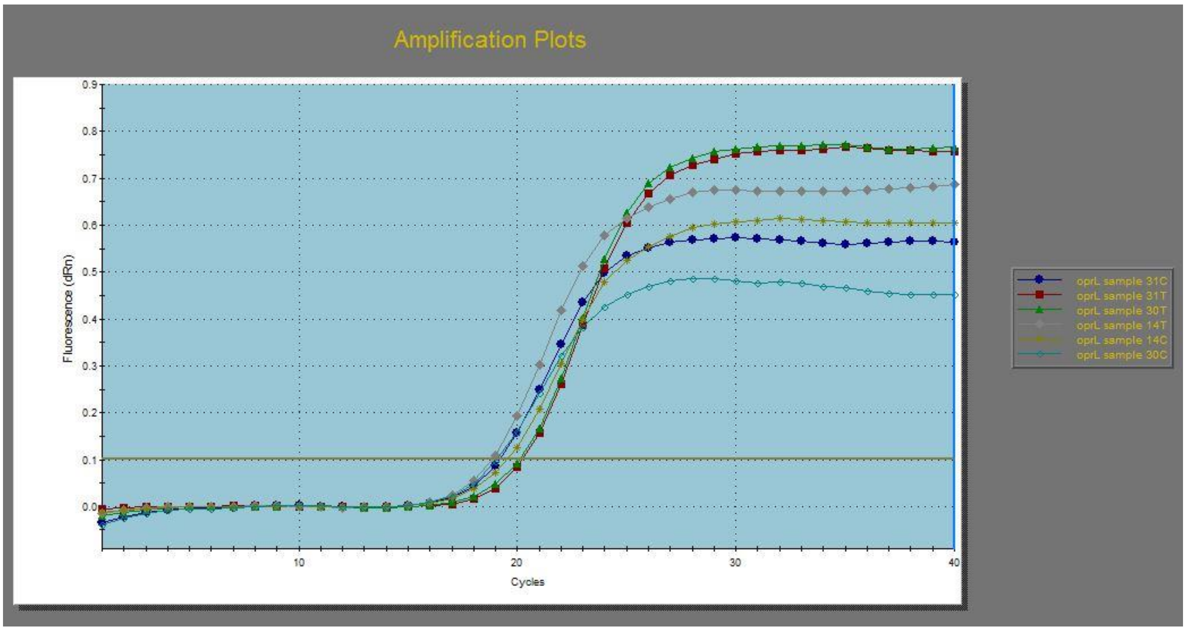

Figure S1: Data sheet of RT-qPCR showing amplification curves of *MexA* (**A**) *MexB* (**B**) and *OprL* (**C**) genes of *P. aeruginosa*.
